# Fork PCR: a universal and efficient genome-walking tool

**DOI:** 10.3389/fmicb.2023.1265580

**Published:** 2023-09-22

**Authors:** Hao Pan, Xinyue Guo, Zhenkang Pan, Rongrong Wang, Bingkun Tian, Haixing Li

**Affiliations:** ^1^School of Chemistry and Chemical Engineering, Nanchang University, Nanchang, China; ^2^State Key Laboratory of Food Science and Resources, Nanchang University, Nanchang, China; ^3^Sino-German Joint Research Institute, Nanchang University, Nanchang, China

**Keywords:** genome-walking, fork primer set, primer overlap, randomly partial annealing, nested PCR

## Abstract

The reported genome-walking methods still suffer from some deficiencies, such as cumbersome experimental steps, short target amplicon, or deep background. Here, a simple and practical fork PCR was proposed for genome-walking. The fork PCR employs a fork primer set of three random oligomers to implement walking task. In primary fork PCR, the low-stringency amplification cycle mediates the random binding of primary fork primer to some places on genome, producing a batch of single-stranded DNAs. In the subsequent high-stringency amplification, the target single-strand is processed into double-strand by the site-specific primer, but a non-target single-stranded DNA cannot be processed by any primer. As a result, only the target DNA can be exponentially amplified in the remaining high-stringency cycles. Secondary/tertiary nested fork PCR(s) further magnifies the amplification difference between the both DNAs by selectively enriching target DNA. The applicability of fork PCR was validated by walking several gene loci. The fork PCR could be a perspective substitution for the existing genome-walking schemes.

## 1. Introduction

Genome-walking is a strategy to reveal unknown flanking segments according to known DNA sequences. The application scenarios of genome-walking include but are not limited to (i) isolating microbes (Li et al., 2023; Wei et al., 2023), (ii) mining new functional genes (Acevedo et al., 2013; Qayyum et al., 2017), and (iii) decoding regulatory sequences of functional genes (Yang et al., 2019; Wang et al., 2022), etc. For example, by using genome-walking, Yamada et al. (2008) characterized the glycosyl hydrolase genes from termite guts and vermiform appendixes of horses; Rajapriya et al. (2021) found a rare T-DNA integration event in rice. Genome-walking has become one of the valuable tools in molecular biology and the associated disciplines, by obtaining information on unknown DNAs of interest. Many progresses in life science are inseparable from this technique (Kotik, 2009; Pei et al., 2022).

So far, there are two types of genome-walking techniques available, including genome library-based technique and PCR-based technique. The former is rarely adopted due to the heavy workload arising from the construction and screening of a genome library. A PCR-based genome-walking method is much more popular (Kotik, 2009; Sun et al., 2022).

The available PCR-based techniques can be clustered into two types: genome pretreatment-dependent PCR (Ochman et al., 1988; Uchiyama and Watanabe, 2006; Yu et al., 2020; Yik et al., 2021) and random priming-based PCR (Liu and Whittier, 1995; Wang et al., 2007; Sun et al., 2022). The pretreatment is mainly composed of the restriction cleavage of genome and the subsequent ligation operation. This pretreatment not only increases workload but also extends experimental period (Sun et al., 2022; Wang et al., 2022). Most PCR-based walking techniques, such as ligation PCR, panhandle PCR, and inverse PCR, falls into the former type (Robinson et al., 2006; Uchiyama and Watanabe, 2006; Yik et al., 2021). The latter type is completely free of genomic template pretreatment preceding PCR. As a result, random priming-based PCR has occupied a major position in genome-walking (Chang et al., 2018; Wang et al., 2023).

A few random priming-based PCR methods, such as wristwatch PCR (Wang et al., 2022), fusion primer driven racket PCR (Pei et al., 2022), and differential annealing-mediated racket PCR (Sun et al., 2022), have been constructed during the past decades. The experimental processes of these PCRs may be diverse, but the involved working principles are similar. All these PCRs without exception rely on at least one low-stringency amplification cycle that encourages walking primer to partially anneal to flank DNA, thus achieve so-called walking. In these methods, three rounds of one-sided nested PCRs are generally required to obtain an acceptable level of amplification specificity (Kotik, 2009; Pei et al., 2022; Sun et al., 2022; Wang et al., 2022). In addition, these PCRs still suffer from some shortcomings, such as severe background, heavy workload, or short target amplicon. These shortcomings are usually related to the low-stringency cycle(s) performed in each round of PCR (Liu and Whittier, 1995; Li et al., 2015; Chang et al., 2018). How to overcome these shortcomings as much as possible is the chief challenge in devising a genome-walking method.

In the current work, a new genome-walking approach termed fork PCR is proposed. This PCR depends on the partial overlaps between a fork primer set of three primers. The feasibility of fork PCR was verified by walking three genes in *Levilactobacillus brevis* CD0817 (Gao et al., 2019) and one gene in rice (Wang et al., 2022).

## 2. Materials and methods

### 2.1. Genomic DNAs

The cells of *L. brevis* CD0817 were propagated as reported earlier (Jia et al., 2022; Li et al., 2022). The microbial genomic DNA was separated from the cells using the TIANamp Bacteria DNA Kit (Tiangen Biotech Co., Ltd., Beijing, China) in accordance to the instruction. Rice genomic DNA was given by Dr. Xiaojue Peng working at Nanchang University (Nanchang, China).

### 2.2. Primers

**FIGURE 1:**
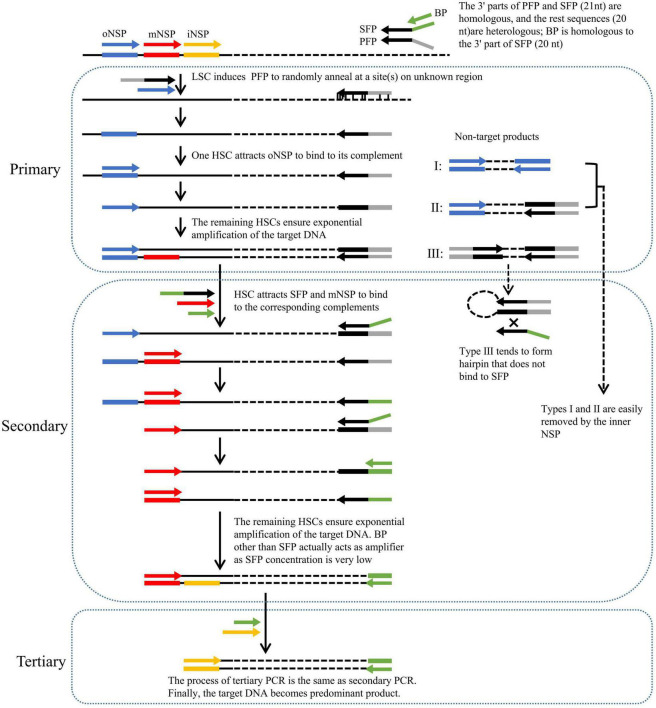
Schematic of fork PCR. oNSP, mNSP, and iNSP mean outmost, middle, and innermost nested site-specific primers, respectively. PFP, SFP, and BP mean primary fork primer, secondary fork primer, and branch primer, respectively. Solid lines show known DNA, and dotted lines show unknown DNA. Arrows represent primers, and rectangles of the same color as arrows represent primer complements. HSC and LSC are the abbreviations of high-stringency cycle and low-stringency cycle, respectively.

A fork primer set, consisting of three random primers [primary fork primer (PFP), secondary fork primer (SFP), and branch primer (BP)], was devised to perform walking task. The PFP or SFP is 41 nt in length, consisting of 3′ stem of 21 nt and 5′ branch of 20 nt. The stems of PFP and SFP are homologous, while the branches are heterologous. The BP corresponds to the branch of SFP (Figure 1). The Tm values of the stems or branches lie between 60 and 65°C. The Tm value of full-length PFP or SFP is rather high (approximate 75°C). In this study, two fork primer sets were designed, aiming to do parallel PCRs in a genome-walking experiment. The two SFPs have an identical BP part, that is, the BP is universal to the two secondary PCRs or tertiary PCRs. Three nested site-specific primers (NSP), outmost NSP (oNSP), middle NSP (mNSP), and innermost NSP (iNSP), were selected from each given gene along the direction of 5′ to 3′. The three NSPs show near Tm values (60–65°C) to those of the PFP/SFP stems or branches. Each primer itself or primer pair avoids generating severe hairpins or dimers (Table 1). Moreover, the four bases (A, T, C, and G) should be evenly distributed in a primer as possible; and a situation of consecutive four identical bases must be avoided. The whole genome sequence of *L. brevis* CD0817 is available at GenBank with accession no. CP032931.1 (Gao et al., 2019). The *hyg* along with the surrounding sequence please refer to accession no. KF206149.1 (Wang et al., 2022).

**TABLE 1:** Primers used in this study.

Primer set^§^	Primary PCR	Secondary PCR	Tertiary PCR
Fork 1	PFP1: ACGCGTAATAGCTCGGGATG ATGCTGCTCGTGGATGACTCT	SFP1: CCTGACCGCCTTCTACACC TATGCTGCTCGTGGATGACTCT BP: CCTGACCGCCTTCTACACCT	BP: CCTGACCGCCTTCTACACCT
Fork 2	PFP2: ATCCGCCCATAGCCTTCAGT GACTACGCTGCCTTGCTACTT	SFP2: CCTGACCGCCTTCTACACCTGA CTACGCTGCCTTGCTACTT BP: CCTGACCGCCTTCTACACCT	
*gadA*	GTTTCTGGTCACAAGTACGGCATGG	TGCTGATACGCTGCCAGAAGAAATG	ACGGTTGACTCCATTGCCATTAACT
*gadR*	TCCTTCGTTCTTGATTCCATACCCT	CCATTTCCATAGGTTGCTCCAAGG	GGATACTGGCTAAAATGAATTAACTCGGATAA
*pct*	TCTTGTTCTTCAACAGTGGTGGGTA	TCGTCTTTCGTGTAAGTGTTGGTGT	AGGAAATATGCACTCTTGGGAAGCG
*hyg*	ACGGCAATTTCGATGATGCAGCTTG	GGGACTGTCGGGCGTACACAA	CTGGACCGATGGCTGTGTAGAAG

^§^The two fork primer sets were, respectively paired with a NSP (nested site-specific primer) set to perform two parallel sets of fork PCRs. A PFP (primary fork primer) was paired with a NSP in the same column for a primary PCR. A SFP (secondary fork primer) and the BP (branch primer) were paired with a NSP in the same column for a secondary PCR. The BP was paired with a NSP in the same column for a tertiary PCR. The BP is universal to the two secondary PCRs or tertiary PCRs.

### 2.3. PCR procedure

Primary fork PCR was proceeded by PFP and oNSP, using genomic DNA as template. The PCR reaction components (10–100 ng microbial genome or 100–1,000 ng rice genome, 0.4 mM each dNTP, 0.2 μM PFP, 0.2 μM oNSP, 1 × LA Taq buffer II plus 2.5 mM Mg^2+^, and 2.5 U TaKaRa LA Taq polymerase) were assembled into a 200 μL PCR tube and replenished to 50 μL with water.

Secondary fork PCR was proceeded by SFP, BP, and mNSP, using primary PCR product as template. The PCR reaction components [1 μL primary PCR product (dilute 10–1,000 folds if necessary), 0.4 mM each dNTP, 0.02 μM SFP, 0.2 μM BP, 0.2 μM mNSP, 1 × LA Taq buffer II plus 2.5 mM Mg^2+^, and 2.5 U TaKaRa LA Taq polymerase] were assembled into a 200 μL PCR tube and replenished to 50 μL with water.

Tertiary PCR was proceeded by BP and iNSP, using secondary PCR product as template. The PCR reaction components [1 μL secondary PCR product (dilute 10–1,000 folds if necessary), 0.4 mM each dNTP, 0.2 μM BP, 0.2 μM iNSP, 1 × LA Taq buffer II plus 2.5 mM Mg^2+^, and 2.5 U TaKaRa LA Taq polymerase] were assembled into a 200 μL PCR tube and replenished to 50 μL with water.

Primary fork PCR was completed by thirty high-stringency annealing cycles following one low-stringency annealing cycle. While secondary/tertiary PCR was completed by thirty high-stringency annealing cycles. The detailed parameters of the PCR reactions are shown in Table 2.

**TABLE 2:** Thermal cycling parameters used in fork PCR.

Round of PCR	Thermal parameters	Cycle number
Primary	95°C, 2 min	
	95°C, 10 s; 25°C, 30 s; 72°C, 2 min	1
	95°C, 10 s; 65°C, 30 s; 72°C, 2 min	30
	72°C, 5 min	
Secondary	95°C, 2 min	
	95°C, 10 s; 65°C, 30 s; 72°C, 2 min	30
	72°C, 5 min	
Tertiary	Identical to the secondary PCR	

### 2.4. Agarose electrophoresis and DNA sequencing

The PCR products were separated exploiting electrophoresis on 1% agarose gel. The major secondary/tertiary amplicons were recovered using the DiaSpin DNA Gel Extraction kit (Sangon Biotech Co., Ltd., Shanghai, China), and their sequencing were completed by Sangon Biotech Co., ltd. (Shanghai, China). The sequencing results were assessed using the Lasergene software (DNASTAR, Inc.).

## 3. Results

### 3.1. Principle of fork PCR

The key to fork PCR is the design of a fork primer set of three primers PFP, SFP, and BP. The 3′ part of PFP overlaps the 3′ part (21 nt) of SFP, while BP overlaps the 5′ part (20 nt) of SFP. The guidelines for designing fork primer set are presented in the Section of “2.2 Primers.” The three primers form a fork-like structure (Figure 1). The current method is thus nominated as fork PCR.

Primary fork PCR is driven by both PFP and oNSP. The initial low-stringency annealing cycle heartens PFP to partially bind to multiple sites on genome, generating a batch of first-strand DNAs that are constituted by target and non-target ones. The subsequent high-stringency cycle exclusively enables oNSP to recognize its complement on the target first-strand, guiding the synthesis of the target second-strand. This target second-strand is exponentially boosted in the next high-stringency cycles, as it owns oNSP at the 5′ end and PFP complement at the 3′ end. Rather, a non-target first-strand cannot be further processed, as it has no perfect binding for any primer. Despite all this, three categories of unwanted amplicons may be still produced, namely, those produced from oNSP alone (I), those produced from both oNSP and PFP (II), and those produced from PFP alone (III), respectively.

Secondary fork PCR is conducted by SFP, BP, and mNSP under the stringent conditions, using the primary PCR reaction solution as template. Please note, the concentration of SFP is just 1/10 of that of BP or mNSP. In the first PCR cycle, SFP is oriented to the PFP site at the 3′ end of primary target product, and thereafter extends toward known region. Consequently, a strand, with SFP at the 5′ end and mNSP complement at the 3′ end, is thus obtained. In the remaining cycles, this strand undergoes an exponential amplification like a classical endpoint PCR by mNSP and BP (because the concentration of SFP is very low, BP rather than SFP actually participates in amplification). The major role of SFP is to integrate BP into PFP site. Conversely, categories I and II non-target primary products are readily diluted by mNSP, because they have no authentic complementary site for this primer. Type III non-target product is more likely to form hairpin on its own, rather than anneal with primer SFP or BP, due to the fact that its PFP inverted terminal is much longer than the homologous part between PFP and SFP or BP.

Tertiary fork PCR is performed by iNSP and BP, using the secondary PCR reaction solution as template. The working mechanism of tertiary PCR is consistent with secondary PCR. Eventually, the target DNA is highlighted.

### 3.2. Effects of SFP concentration on secondary fork PCR

**FIGURE 2:**
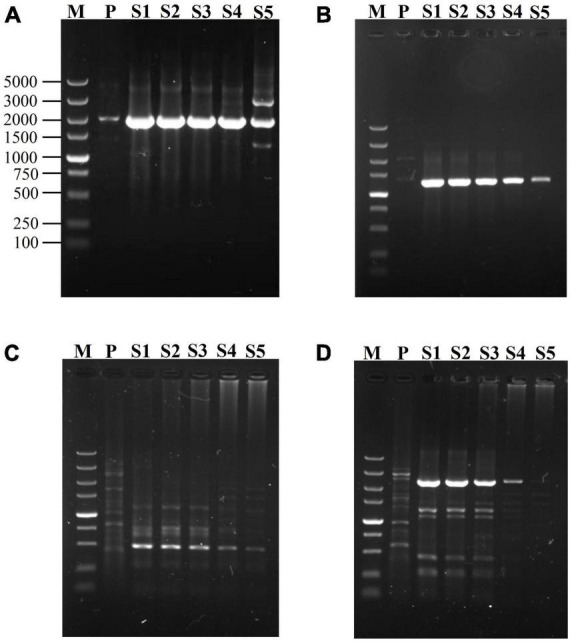
Effects of SFP concentration on secondary fork PCR. mNSP (middle nested site-specific primer) and BP (branch primer) were used at 0.2 μM, but SFP (secondary fork primer) concentration was varied (lanes S1-S5 indicate 0.2, 0.02, 0.002, 0.0002, 0.00002 μM SFP in sequence). Secondary amplifications of *gadA*, respectively participated by SFP1 **(A)** and by SFP2 **(B)**; and secondary amplifications of *hyg*, respectively participated by SFP1 **(C)** and by SFP2 **(D)**. Lane M: TaKaRa DL5000 Marker; and lane P: primary PCR product.

Since SFP plays a transformation role between the first two rounds of PCRs, the effect of its concentration on the reaction is possibly a worthwhile question. The genes of *gadA* and *hyg* were used to evaluate the influences of SFP concentration on secondary fork PCR. The two SFPs were simultaneously tested at various concentrations (0.2, 0.02, 0.002, 0.0002, and 0.00002 μM), with both mNSP and BP being fixed at 0.2 μM. As is shown in Figure 2, 0.002–0.2 μM of SFP resulted in good and similar results for the both genes. To be more specific, when SFP ranged from 0.002 to 0.2 μM, the brightness of a major target DNA band in an electropherogram were almost the same; and when SFP was below 0.002 μM, the brightness was gradually weakened with decreasing the concentration of SFP. Herein, 0.02 μM SFP was chosen.

### 3.3. Validation of fork PCR

**FIGURE 3:**
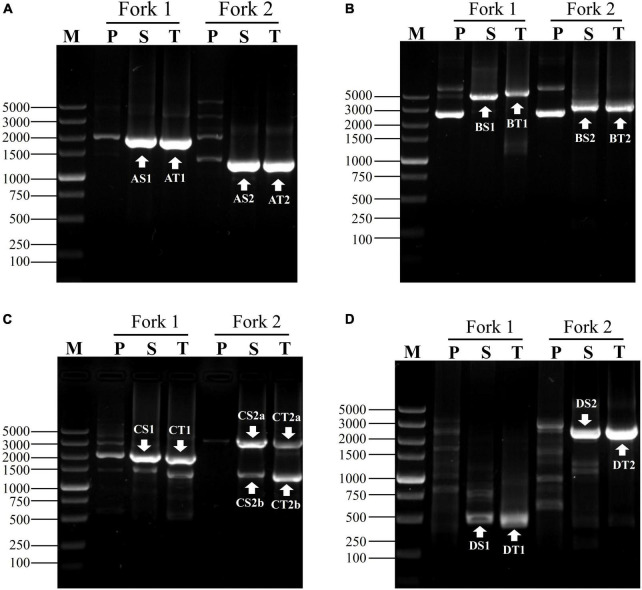
Exploring adjacent sequences of genes of **(A)** *gadA*, **(B)** *gadR*, **(C)** *pct*, and **(D)** *hyg*. Fork 1 and fork 2 denote the two parallel fork PCRs in each walking experiment. The fragments marked with white arrows indicate the target products. Lanes P, S, and T show primary, secondary, and tertiary PCR, respectively. Lane M was the TaKaRa DL5000 Marker.

To attest the applicability of fork PCR, we employed this method to identify segments flanking *pct*, *gadA* and *gadR* genes in *L. brevis* CD0817 and *hyg* gene in rice. A NSP set of three primers (oNSP, mNSP, and iNSP) were chosen from each gene site (Table 1). Each NSP set was paired with two fork primer sets, respectively, to complete the walking. The PCR conditions are presented in Table 2. Upon the completion of the PCRs, all the primary, secondary, and tertiary PCR products were electrophoresed and visualized on 1% agarose gel. The results are illustrated in Figure 3. As is shown, each secondary/tertiary amplification released one to two clear band, with negligible background. Sequencing to these bands demonstrated that they all were wanted products, because the NSP-sided part of each product overlapped that of the corresponding known region. The size of the target amplificons lied between 0.5 and 5.0 kb. And the largest DNA fragments retrieved in each walking experiment ranged from 2.0 to 5.0 kb.

## 4. Discussion

To date, numerous PCR-based genome-walking protocols have been published (Liu and Whittier, 1995; Strauss et al., 2001; Wang et al., 2007; Lee et al., 2011; Thanh et al., 2012). A method that does not require the pretreatment of genomic DNA template has been always favored (Pei et al., 2022; Wang et al., 2022). The recent devised fusion primer driven racket PCR (Pei et al., 2022), wristwatch PCR (Wang et al., 2022), and differential annealing-mediated racket PCR (Sun et al., 2022) are such a method. However, these methods are generally susceptible to at least one of the following the disadvantages: time-consuming, low efficiency, or unacceptable specificity (Li et al., 2015; Wang et al., 2022). It is attractive to develop a genome-walking method overcoming these disadvantages (Wang et al., 2022; Lin et al., 2023). The proposed fork PCR based on random priming is such a strategy.

The fork PCR was partially inspired by the bridging PCR. In the bridging method secondary PCR, once bridging primer anneals to primary walking primer site, the site will elongate along the bridging primer to produce a DNA complementary to this primer. As a result, an undesired primary product defined by the walking primer alone will be amplified by this bridging primer (Lin et al., 2023). In the current fork PCR, however, the 5′ parts of SFP and PFP are heterologous, hindering PFP site from elongating along SFP. Clearly, an undesired primary product originated from PFP alone has little chance to be amplified by SFP/BP in secondary PCR.

The fork PCR shows a satisfactory amplification specificity. This satisfactory specificity is mainly attributed to the special design of a fork primer set. The fork primer set efficiently enriches target DNA like the classical endpoint PCR, meanwhile suppresses non-target product amplification. Due to the genomic complexity and the low-stringency cycle, three non-target products may be expected in primary PCR: (I) those initiated by oNSP alone; (II) those initiated by both PFP and oNSP; and (III) those initiated by PFP alone (Liu and Whittier, 1995; Wang et al., 2022; Lin et al., 2023). Categories I and II non-targets will be readily removed by the inner NSP in next PCR. The real challenge is how to eliminate category III non-target (Liu and Whittier, 1995; Kotik, 2009; Wang et al., 2022; Lin et al., 2023). In this study, we introduced suppression PCR for this aim. It has been verified that a fragment confined by an inverted repeat more than 40 nt prefers to form hairpin, rather than binds with a primer just corresponding to the 5′ part of this inverted repeat (Siebert et al., 1995; Chenchik et al., 1996). In this work, we set the PFPs and SFPs to be 41 nt, which have a 21 nt of 3′ overlap. As a result, in secondary PCR, type III non-target DNA itself is prone to forming hairpin mediated by PFP terminal, instead of hybridizing with SFP. Even if SFP occasionally anneals to PFP site on category III non-target, this non-target cannot be efficiently amplified by SFP because the concentration of SFP is very low. SFP is only responsible for bridging BP into PFP site. And the real amplifier BP is much shorter than SFP (Siebert et al., 1995; Chenchik et al., 1996; Lin et al., 2023). Clearly, type III non-target cannot be amplified in this fork PCR.

The amplification specificity of fork PCR is also associated with the following fact. The secondary/tertiary fork PCR is entirely composed of high-stringency annealing cycles, which is helpful for ensuring the specificity of this method. The other genome-walking PCRs, however, have to perform at least one secondary/tertiary reduced-stringency cycle (Liu and Whittier, 1995; Li et al., 2015; Chang et al., 2018). For instance, the wristwatch PCR relies on a wristwatch primer set of three primers, which form a wristwatch-like structure under about 40°C. Thus, a reduced-stringency cycle must be conducted in secondary or tertiary PCR, encouraging the wristwatch primer to hybridize to the previous wristwatch primer site. At the same time, however, this cycle also contributes to non-target amplification; and, it may cause the primer to anneal inside a target DNA, producing a smaller product(s) (Wang et al., 2022, 2023).

The fork PCR exhibits a high success rate. This arises from the two inherent features of the method. First, the primary low-stringency (25°C) cycle should aid PFP to find a binding site(s) on DNA of interest. In general, walking experiment will succeed once this binding occurs (Chang et al., 2018; Wang et al., 2022). Second, parallel fork PCR sets can be arranged for a genome-walking. It is expected that at least one fork PCR set can generate a positive walking outcome if parallel ones are performed. Based on the walking rationale, the success rate of fork PCR is anticipated to be equal to that of wristwatch PCR (Wang et al., 2022, 2023).

SFP acts as a bridge between primary product and BP in secondary PCR. In other words, SFP is only responsible for integrating BP into PFP site, and the actual amplifier is BP. Therefore, a much lower concentration of SFP than BP is expected (Chenchik et al., 1996; Lin et al., 2023). However, our experimental data indicated that SFP works well at the same concentration (0.2 μM) as BP. We attribute this to the fact that there is only a short overlap between SFP and PFP. By comprehensive consideration, we suggest 0.02 μM of SFP (1/10 concentration of BP or mNSP) in the fork PCR, so as to exclude any potential amplification of SFP on category III non-target product.

Tertiary amplification is generally unnecessary, since all the secondary fork PCRs gave a positive result. The aforementioned specificity guarantee design for fork PCR rationalizes the unnecessity of tertiary amplification. This feature facilitates fork PCR rapidly acquiring walking data (Wang et al., 2022, 2023). In this regard, fork PCR method surpasses most the existing methods that have to proceed three rounds of amplifications.

## 5. Conclusion

In this work, a versatile, accurate, and simple fork PCR approach was devised for genome-walking. This PCR relies on the fork-like structure formed by a walking primer set of three primers. Performing two rounds of amplification reactions suffices to give out positive outcome. The fork PCR-based genome-walking is beneficial to saving time and cost. The applicability of fork PCR was validated by walking several gene loci from *Levilactobacillus brevis* CD0817 and rice. This proof-of-concept novel approaches could be a perspective alternative to the earlier genome-walking schemes.

## Data availability statement

The original contributions presented in this study are included in the article/supplementary material, further inquiries can be directed to the corresponding author.

## Author contributions

HP: Investigation, Visualization, Writing—original draft. XG: Writing—original draft, Data curation. ZP: Project administration, Writing—original draft. RW: Writing—review and editing, Resources. BT: Writing—review and editing, Formal analysis. HL: Writing—review and editing, Conceptualization, Funding acquisition.
